# Candida auris Bloodstream Infection Induces Upregulation of the PD-1/PD-L1 Immune Checkpoint Pathway in an Immunocompetent Mouse Model

**DOI:** 10.1128/msphere.00817-21

**Published:** 2022-02-28

**Authors:** Sebastian Wurster, Nathaniel D. Albert, Dimitrios P. Kontoyiannis

**Affiliations:** a Department of Infectious Diseases, Infection Control and Employee Health, The University of Texas M.D. Anderson Cancer Center, Houston, Texas, USA; University of Georgia

**Keywords:** *Candida auris*, animal model, host response, immune checkpoint pathways, inflammation

## Abstract

Candida auris is a globally spreading yeast pathogen causing bloodstream infections with high mortality in critically ill patients. The inherent antifungal drug resistance of most C. auris isolates and threat of multidrug-resistant strains create a need for adjunct immunotherapeutic strategies. While C. albicans candidemia was shown to induce immune paralysis and activation of inhibitory immune checkpoints, *in vivo* data on host responses to C. auris bloodstream infection are lacking as is an immunocompetent murine infection model to study the immunopathology and immunotherapy of C. auris sepsis. Therefore, herein, we developed an immunocompetent C. auris sepsis model by intravenously infecting C57BL/6 mice with 1.5 × 10^8^ to 8 × 10^8^ yeast cells of aggregate-forming (AR-0384) and nonaggregative (AR-0381) C. auris reference isolates. Both isolates caused reproducible, inoculum-dependent increasing morbidity, mortality, and fungal burden in kidney tissue. Notably, morbidity and mortality outcomes were partially decoupled from fungal burden, suggesting a role of additional modulators of disease severity such as host immune responses. Flow cytometric analyses of splenic immune cells revealed significant upregulation of the programmed cell death protein 1 (PD-1) on T cells and its ligand PD-L1 on macrophages from mice infected with C. auris AR-0384 compared to uninfected mice. PD-L1 expression on macrophages from AR-0384-infected mice strongly correlated with fungal tissue burden (Spearman’s rank correlation coefficient [ρ] = 0.95). Altogether, our findings suggest that C. auris sepsis promotes a suppressive immune phenotype through PD-1/PD-L1 induction, supporting further exploration of PD-1/PD-L1 blockade as an immunotherapeutic strategy to mitigate C. auris candidiasis.

**IMPORTANCE** Health authorities consider Candida auris to be one of the most serious emerging nosocomial pathogens due to its transmissibility, resistance to disinfection procedures, and frequent antifungal drug resistance. The frequency of multidrug-resistant C. auris isolates necessitates the development of novel therapeutic platforms, including immunotherapy. However, *in vivo* data on host interactions with C. auris are scarce, compounded by the lack of reliable immunocompetent mammalian models of C. auris candidemia. Herein, we describe a C. auris sepsis model in immunocompetent C57BL/6 mice and demonstrate reproducible and inoculum-dependent acute infection with both aggregate-forming and nonaggregative reference isolates from different clades. Furthermore, we show that C. auris sepsis induces upregulation of the PD-1/PD-L1 immune checkpoint pathway in infected mice, raising the potential of a therapeutic benefit of immune checkpoint blockade. Our immunocompetent model of C. auris sepsis could provide a facile preclinical platform to thoroughly investigate immune checkpoint blockade and combination therapy with antifungals.

## OBSERVATION

The globally emerging yeast pathogen Candida auris (1–2) represents a unique threat due to its ability to cause bloodstream infections in critically ill patients, its transmissibility in health care environments, and its ability to withstand disinfection procedures (3–4). Of concern, a significant proportion of C. auris strains worldwide are resistant to multiple, and sometimes even to all, available classes of antifungal drugs (3–6). Therefore, adjunct therapeutic strategies, including immunotherapy, are increasingly investigated (7).

While the immune pathogenesis of C. auris is so far poorly characterized, prior research showed that Candida albicans sepsis promotes a paralytic immune status characterized by expression of suppressive immune checkpoint pathways, functional impairment of lymphocytes and phagocytes, and anti-inflammatory cytokine responses (8). These features of immune paralysis are often associated with poor outcomes despite appropriate antifungal therapy, increased risk of secondary infections, and long-lasting immune impairment in survivors (8–9). In contrast to C. albicans candidemia, such immunological data for C. auris bloodstream infection are lacking as are immunocompetent murine C. auris infection models to study host responses and immunotherapies. Therefore, we herein optimized a C. auris bloodstream infection model in immunocompetent C57BL/6 mice and studied the activation of coinhibitory immune checkpoint molecules in splenic immune cells of infected mice.

Inocula of two C. auris reference isolates from the U.S. Centers for Disease Control and Prevention antimicrobial resistance (AR) isolate bank (10), the nonaggregative clade II isolate AR-0381 and the aggregative clade III isolate AR-0384, were prepared as described in Text S1 in the supplemental material. Eight-week-old female C57BL/6 mice received an injection of approximately 1.5 × 10^8^, 4 × 10^8^, or 8 × 10^8^ yeast cells into the lateral tail vein. Survival was monitored daily for 7 days postinfection. Morbidity was scored on day +7 using the modified murine sepsis score (MSS) (11), which ranges from 0 (no signs of distress) to 3 (moribund). Animals that died before day +7 received a score of 4. Fungal burden in left kidneys as a sentinel organ (12) was quantified as described before (13–14), with minor modifications detailed in Text S1. All procedures were reviewed and approved by MD Anderson’s institutional animal care and use committee (protocol number 00002065-RN00).

10.1128/mSphere.00817-21.1 TEXT S1 Detailed descriptions of experimental procedures for preparation of Candida auris inoculums and infection of mice, determination of fungal burden in kidney tissue, isolation of murine splenocytes, and fluorescent labelling of splenocytes. Download Text S1, DOCX file, 0.03 MB. Copyright © 2022 Wurster et al. 2022 Wurster et al. https://creativecommons.org/licenses/by/4.0/ This content is distributed under the terms of the Creative Commons Attribution 4.0 International license.

**FIG 1:**
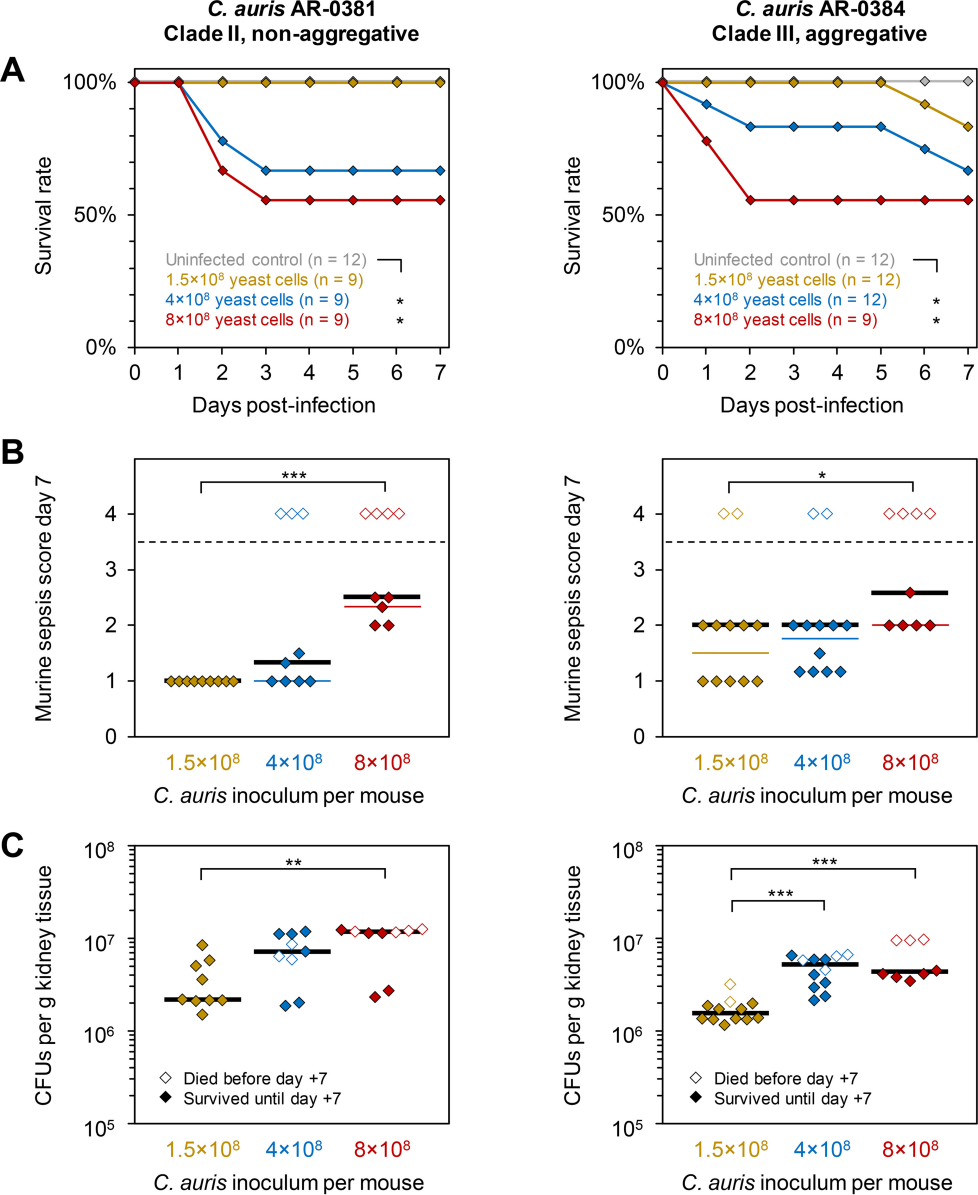
Establishment of reproducible C. auris infection in immunocompetent C57BL/6 mice. Eight-week-old, immunocompetent female C57BL/6 mice were intravenously injected with approximately 1.5 × 10^8^, 4 × 10^8^, or 8 × 10^8^ yeast cells of C. auris isolates AR-0381 or AR-0384. For each strain, two independent experiments were performed. Aggregated results are shown. (A) Survival curves were compared using the log rank test. (B) Distributions of 7-day murine sepsis scores (MSS) were used as a measure of infection severity. Black bars indicate medians among all mice. Thin colored bars indicate medians among survivors. (C) Numbers of C. auris CFU were quantified by streaking kidney tissue homogenates on Sabouraud dextrose agar upon natural death or on day 7 postinfection. Black bars = medians. (B and C) Kruskal-Wallis test with Dunn’s multiple comparison test. *, *P* < 0.05; **, *P* < 0.01; ***, *P* < 0.001.

Infection of immunocompetent mice with 1.5 × 10^8^ AR-0381 cells remained sublethal, and the mice developed only mild signs of distress (median day 7 MSS, 1.0) (Fig. 1A and B). In contrast, higher inocula (4 × 10^8^ and 8 × 10^8^) of AR-0381 cells caused increasing morbidity (median MSS, 1.3 and 2.5) and 7-day mortality (33% and 44%) (Fig. 1A and B). The trend of inoculum-dependent morbidity persisted when restricting the analysis to mice that survived until day +7 (Fig. 1B). Likewise, fungal burden steadily increased at higher inocula, with 2.2 million, 7.2 million, and 11.3 million CFU recovered per gram of kidney tissue from mice infected with 1.5 × 10^8^, 4 × 10^8^, and 8 × 10^8^ AR-0381 cells, respectively (Fig. 1C).

Unlike strain AR-0381, 1.5 × 10^8^ yeast cells of the aggregate-forming isolate AR-0384 caused robust morbidity (median MSS, 2.0; median among survivors, 1.5) and 17% 7-day mortality (Fig. 1A and B). Infection outcomes further worsened in mice infected with 4 × 10^8^ AR-0384 cells, with 33% 7-day mortality and a median MSS of 2.0 (median among survivors, 1.8) (Fig. 1A and B). Infection with 8 × 10^8^ AR-0384 cells caused considerable early mortality and severe distress in surviving animals (median MSS, 2.6; median among survivors, 2.0) (Fig. 1A and B). However, across all three inocula, animals infected with strain AR-0384 displayed lower median fungal kidney burden than AR-0381-infected mice, with 1.5 million, 5.2 million, and 4.3 million CFU per gram kidney tissue, respectively (Fig. 1C). This trend was confirmed by quantitative PCR (15), suggesting that lower fungal burden in AR-0384-infected mice is not due to differences in plating efficiency (data not shown).

Overall, our results are consistent with prior work suggesting that immunocompetent mice display relatively strong resilience to C. auris infection (16). Nonetheless, high-inoculum bloodstream infection with two isolates from different clades and with disparate aggregative capacity elicited severe and partially lethal disease in our model. Interestingly, the relative morbidity/mortality caused by the two strains was partially decoupled from fungal burden, suggesting a role of additional modulators of disease severity such as disparate host responses. Several previous *in vitro* and *in vivo* studies yielded divergent (strain- and inoculum-dependent) results regarding the comparative pathogenicity and immunopathology of aggregate-forming and nonaggregative C. auris strains (6, 13, 17–19). Notably, an aggregative C. auris isolate elicited stronger inflammation and cytotoxicity than a nonaggregative strain in a recently published human epithelial wound model (19). Although clade-specific differences in pathogenicity are a possible confounder in both the cited work and our present study, adverse host responses might contribute to the immunopathology of aggregative C. auris strains and, potentially, worse infection outcomes.

**FIG 2:**
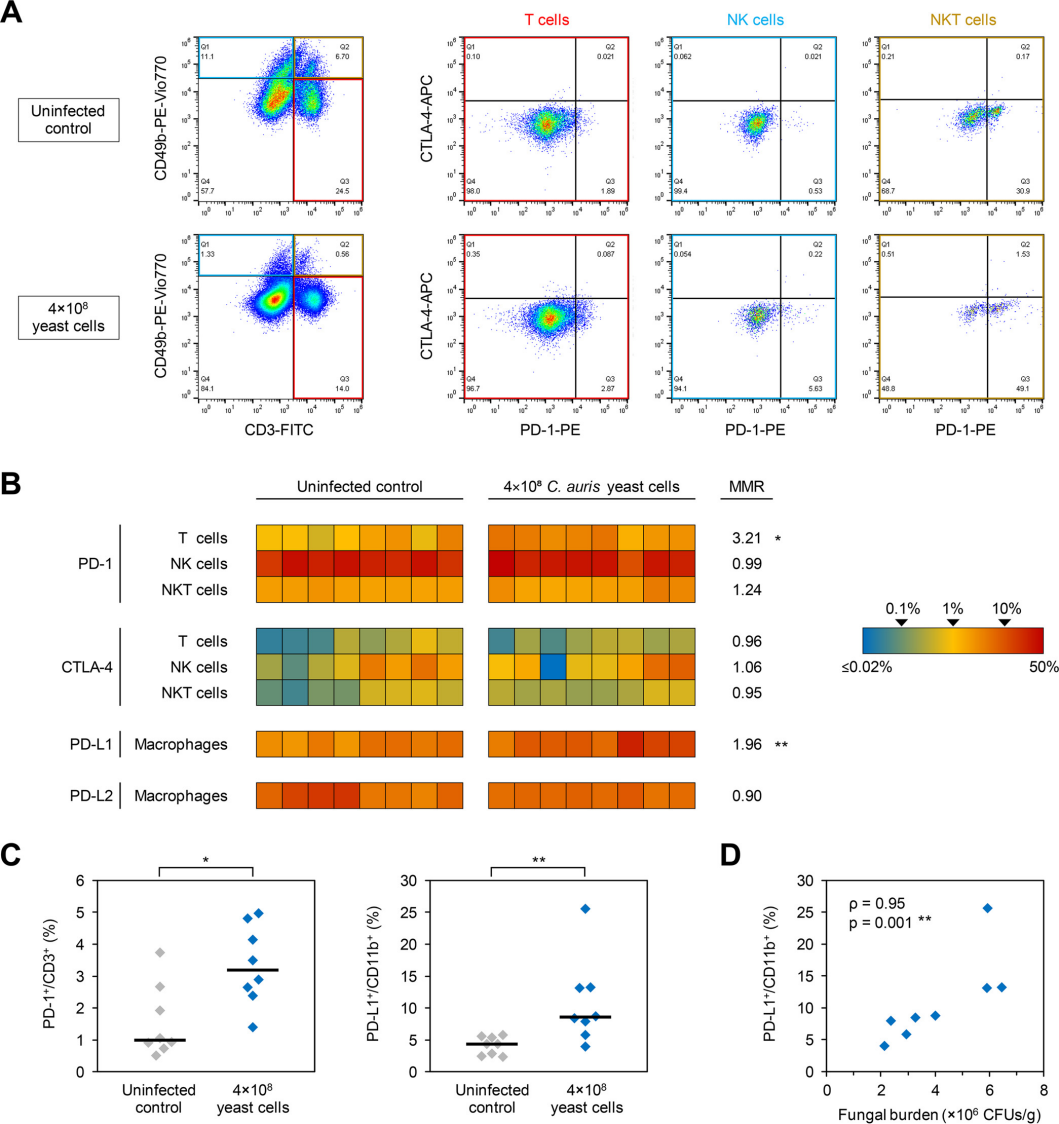
C. auris sepsis induces upregulation of the PD-1/PD-L1 axis. Splenic immune cells were isolated from 8 mice infected for 7 days with 4 × 10^8^ C. auris AR-0384 yeast cells and 8 uninfected control mice. The percentages of PD-1- and CTLA-4-expressing T cells (CD3^+^ CD49b^−^), NK cells (CD3^−^ CD49b^+^), and NKT cells (CD3^+^ CD49b^+^) as well as the percentages of PD-L1- and PD-L2-expressing macrophages (CD11b^+^) were quantified by flow cytometry. (A) Representative data set. (B) Heat map summarizing the individual frequencies of checkpoint marker-positive cells. MMR, median-to-median ratio between infected and uninfected mice. Values above 1.0 indicate higher median frequencies of marker-positive cells in the infected cohort. (C) Distributions of individual and median frequencies (black bars) of PD-1-positive T cells and PD-L1-positive macrophages in C. auris-infected and -uninfected mice. (B and C) Mann-Whitney U test. *, *P* < 0.05; **, *P* < 0.01. (D) Correlation plot comparing the percentage of PD-L1-expressing macrophages (*y* value) with the fungal burden in kidney tissue as a surrogate of infection severity (*x* value). Spearman’s rank correlation coefficient (ρ) and its *P* value are provided. CD, cluster of differentiation; CTLA-4, cytotoxic T-lymphocyte-associated protein 4; PD-1, programmed cell death protein 1; PD-L1/2, programmed death-ligand 1/2.

To test whether C. auris bloodstream infection promotes immune paralysis, especially when due to an aggregative strain, we compared immune checkpoint upregulation in uninfected mice and animals infected with 4 × 10^8^ yeast cells of C. auris strain AR-0384. Therefore, splenocytes were isolated from mice that survived until day +7. The expression of programmed cell death protein 1 (PD-1) and cytotoxic T-lymphocyte-associated protein 4 (CTLA-4) on T cells, natural killer (NK) cells, and natural killer T (NKT) cells was determined by flow cytometry as described in Text S1 in the supplemental material. Representative data are shown in Fig. 2A. C. auris-infected mice harbored significantly higher frequencies of PD-1-positive T cells than uninfected mice (median, 3.2% versus 1.0%; *P* = 0.015) (Fig. 2B and C). In contrast, PD-1 expression on NK and NKT cells as well as CTLA-4 expression on all assayed splenocyte subsets were similar in infected and uninfected mice (median-to-median ratio, 0.99:1.24) (Fig. 2B).

We further tested the expression of the PD-1 ligands PD-L1 and PD-L2 on splenic macrophages. While PD-L2 expression was comparable, significantly higher frequencies of PD-L1-positive macrophages were found in C. auris-infected mice than in controls (median, 8.6% versus 4.4%; *P* = 0.003) (Fig. 2B and C). Interestingly, the percentage of PD-L1-expressing macrophages showed strong positive correlation with the fungal tissue burden (ρ = 0.95, *P* = 0.01) (Fig. 2D). Collectively, these results suggest that C. auris bloodstream infection promotes a suppressive immune phenotype through PD-1/PD-L1 induction, paralleling similar findings in mice (20) and patients (8) with C. albicans candidemia.

The main limitations of this pilot study include testing of only one isolate per aggregation phenotype and a limited number of animals tested per condition. Furthermore, our study focused solely on the induction of coinhibitory immune checkpoint molecules and did not dynamically capture the net state of pro- and anti-inflammatory immune signals in the bloodstream and infected tissues.

Despite these limitations, we herein describe a simple and reproducible C. auris infection model in immunocompetent C57BL/6 mice and demonstrate the induction of coinhibitory immune checkpoint signals after C. auris bloodstream infection. These data provide a theoretical framework for PD-1/PD-L1 blockade as a potential immunotherapeutic strategy to mitigate C. auris candidiasis. We and others previously demonstrated a therapeutic benefit of the PD-1/PD-L1 pathway blockade in murine models of invasive mold infections (21–22) and C. albicans sepsis (20, 23, 24). Our C. auris infection model in cost-efficient C57BL/6 mice could serve as a facile preclinical platform to study checkpoint inhibitors as an investigational therapy for C. auris sepsis. Furthermore, our immunocompetent model could complement the published cyclophosphamide-immunosuppressed and/or neutrophil elastase-deficient murine C. auris sepsis models (6, 16) and allow comparative dynamic immune phenotyping studies to further understand the enigmatic immune pathogenesis of C. auris in different host backgrounds.

10.1128/mSphere.00817-21.2 TABLE S1 Antibodies used for flow cytometry. Antibody solutions were prepared in 100 μL of flow cytometry buffer per sample. Download Table S1, DOCX file, 0.02 MB. Copyright © 2022 Wurster et al. 2022 Wurster et al. https://creativecommons.org/licenses/by/4.0/ This content is distributed under the terms of the Creative Commons Attribution 4.0 International license.
