# Supplementary material for: Candida auris Bloodstream Infection Induces Upregulation of the PD-1/PD-L1 Immune Checkpoint Pathway in an Immunocompetent Mouse Model
Source: mSphere. 2022 Feb 28;7(2):e00817-21. doi: 10.1128/msphere.00817-21 (PMC9044930; doi:10.1128/msphere.00817-21)
Supplement: TEXT S1 [file msphere.00817-21-s0001.docx]

**Supplementary Methods**

*Preparation of Candida auris inoculums and infection of mice*

Yeast peptone dextrose (YPD) agar plates were inoculated with *C. auris* AR-0381 and AR-0384 reference isolates. After incubation at 37 °C for 48 h, single colonies were picked and 5 mL of liquid YPD medium were spiked with the colonies. Cultures were incubated overnight at 35 °C and 200 rpm in a shaking incubator. Thereafter, yeast cells were washed twice with sterile saline, diluted in sterile (infusion-grade) saline, and counted with a hemocytometer. Yeast cell concentrations were adjusted to 1-5×10^9^ yeast cells per mL. Immediately prior to infection of mice, yeast cell suspensions were thoroughly vortexed and passed through a 40-µm cell strainer in order to remove large yeast cell aggregates that might cause embolism. Filtration resulted in a yeast cell loss of 17-26%, as determined by post-filtration counting. Two hundred microliters of the filtered inoculum suspensions were injected with a 30-gauge cannula into the lateral tail vein of 8-week-old female C57BL/6 mice, resulting in final inoculums of approximately 1.5×10^8^, 4×10^8^, and 8×10^8^ yeast cells per mouse.

*Determination of fungal burden in kidney tissue*

After euthanasia of mice by CO_2_ asphyxiation and cervical dislocation, the left kidneys were removed under sterile conditions and weighed on a precision scale. Next, kidneys were placed in a 2.0-mL cryovial containing 1.0 mL of PBS and 15 sterile 3-mm glass beads (Sigma) and the tissue was homogenized with a Mini Bead Beater (Biospec Products). Ten-microliter aliquots of each homogenized kidney sample were added to 990 µL of sterile PBS. One hundred microliters of the dilutions were plated on Sabouraud dextrose agar plates. *C. auris* colonies were counted after incubation for 24 h at 35 °C. The following formula was used to determine the number of colony-forming units (CFUs) per gram tissue:

$$CFUs per g kidney tissue = \frac{number of colonies per mL plated \times dilution factor}{tissue weight \left( g \right) per mL of the homogenate}$$

*Isolation of murine splenocytes*

After euthanasia of mice as described above, spleens were removed aseptically and placed in a 15-mL tube containing 5 mL of ice-cold Roswell Park Memorial Institute medium (RPMI 1640, Gibco) supplemented with 2% fetal bovine serum (FBS). Spleens were then homogenized in 1 mL of RPMI/2% FBS using 1.5-mL biomasher tubes (Nippi). The suspension was passed through a 40-µm cell strainer (Falcon) into a 50-mL tube. After centrifugation for 5 min at 1000×g, the supernatant was discarded, and cells were resuspended in 1 mL of cold erythrocyte lysis buffer (Qiagen). After incubation for 4 min at room temperature, red blood cell lysis was stopped by addition of 14 mL cold RPMI/FBS. After another centrifugation step for 5 min at 1000×g and decantation of the supernatant, the cell pellet was resuspended in 0.5 mL pure ice-cold FBS and transferred to a 1.8-mL cryovial. Thereafter, 0.5 mL of ice-cold freezing medium (80% FBS + 20% DMSO) was added and cells were cryopreserved at -80 °C until further use.

*Fluorescent labelling of splenocytes*

Cryopreserved splenocytes were thawed and slowly added to 10 mL cold flow cytometry buffer (phosphate-buffered saline + 0.5% FBS + 2 mM EDTA). After centrifugation for 5 min at 1000×g, the supernatant was discarded, and the cell pellet was resuspended in 2 mL of flow cytometry buffer. The cell suspension was then passed through a 40-µm cell strainer in order to remove cell aggregates. Cells were quantified with a hemocytometer and the concentration of the cell suspension was adjusted to 1 million cells per mL of flow cytometry buffer. Per sample, two 500-µL aliquots (500,000 cells) were transferred to 2-mL tubes and centrifuged for 5 min at 1000×g. The supernatant was removed, and cells were resuspended in 100 µL antibody solution (**Table S1**). After 20 min incubation at room temperature, cells were washed with 1 mL flow cytometry buffer and centrifuged for 5 min at 1000×g. The supernatant was removed, cells were resuspended in 300 µL of flow cytometry buffer, and cell suspensions were transferred to 5-mL polystyrene round-bottom tubes for measurement. Single-color controls and unstained cells were used for instrument set up and compensation.
