# Supplementary material for: Candida auris Bloodstream Infection Induces Upregulation of the PD-1/PD-L1 Immune Checkpoint Pathway in an Immunocompetent Mouse Model
Source: mSphere. 2022 Feb 28;7(2):e00817-21. doi: 10.1128/msphere.00817-21 (PMC9044930; doi:10.1128/msphere.00817-21)
Supplement: TABLE S1 [file msphere.00817-21-st001.docx]

**Table S1: Antibodies used for flow cytometry.**

| Panel | Marker and fluorochrome | Manufacturer and product number | Volume per sample |
| --- | --- | --- | --- |
| 1 | Anti-CD3-FITC | Miltenyi Biotec, #130-119-798 | 2 µL |
|  | Anti-CD279 (PD1)-PE | Miltenyi Biotec, #130-111-953 | 2 µL |
|  | Anti-CD49b-PE-Vio 770 | Miltenyi Biotec, #130-108-202 | 5 µL |
|  | Anti-CD152 (CTLA-4)-APC | Miltenyi Biotec, #130-116-455 | 2 µL |
| 2 | Anti-CD11b-PE | Miltenyi Biotec, #130-113-806 | 2 µL |
|  | Anti-CD274 (PD-L1)-PE/Cyanine 7 | BioLegend, #124313 | 1.25 µL |
|  | Anti-CD273 (PD-L2)-APC | Miltenyi Biotec, #130-102-879 | 2 µL |

Antibody solutions were prepared in 100 µL of flow cytometry buffer per sample.
